# High‐Speed Embedded Ink Writing of Anatomic‐Size Organ Constructs

**DOI:** 10.1002/advs.202405980

**Published:** 2025-02-11

**Authors:** Weijian Hua, Cheng Zhang, Haoran Cui, Kellen Mitchell, Dale K. Hensley, Jihua Chen, Changwoo Do, Lily Raymond, Ryan Coulter, Erick Bandala, Fazlay Rubbi, Guangrui Chai, Zhengyi Zhang, Yiliang Liao, Danyang Zhao, Yan Wang, Akhilesh K. Gaharwar, Yifei Jin

**Affiliations:** ^1^ Mechanical Engineering Department University of Nevada Reno Reno Nevada 89557 USA; ^2^ State Key Laboratory of High‐Performance Precision Manufacturing Dalian University of Technology Dalian Liaoning 116024 China; ^3^ Center for Nanophase Materials Sciences Oak Ridge National Laboratory Oak Ridge Tennessee 37830 USA; ^4^ Neutron Scattering Division Oak Ridge National Laboratory Oak Ridge Tennessee 37831 USA; ^5^ Department of Industrial and Manufacturing Systems Engineering Iowa State University Ames Iowa 50011 USA; ^6^ Department of Ophthalmology Shengjing Hospital of China Medical University Shenyang Liaoning 110004 China; ^7^ School of Naval Architecture and Ocean Engineering Huazhong University of Science and Technology Wuhan Hubei 430074 China; ^8^ Department of Biomedical Engineering Texas A&M University, College Station Texas 77843 USA

**Keywords:** embedded ink writing, high‐speed printing, organ reconstruction, particle‐hydrogel interactions, yield‐stress fluids

## Abstract

Embedded ink writing (EIW) is an emerging 3D printing technique that fabricates complex 3D structures from various biomaterial inks but is limited to a printing speed of ∼10 mm s^−1^ due to suboptimal rheological properties of particulate‐dominated yield‐stress fluids when used as liquid baths. In this work, a particle‐hydrogel interactive system to design advanced baths with enhanced yield stress and extended thixotropic response time for realizing high‐speed EIW is developed. In this system, the interactions between particle additive and three representative polymeric hydrogels enable the resulting nanocomposites to demonstrate different rheological behaviors. Accordingly, the interaction models for the nanocomposites are established, which are subsequently validated by macroscale rheological measurements and advanced microstructure characterization techniques. Filament formation mechanisms in the particle‐hydrogel interactive baths are comprehensively investigated at high printing speeds. To demonstrate the effectiveness of the proposed high‐speed EIW method, an anatomic‐size human kidney construct is successfully printed at 110 mm s^−1^, which only takes ∼4 h. This work breaks the printing speed barrier in current EIW and propels the maximum printing speed by at least 10 times, providing an efficient and promising solution for organ reconstruction in the future.

## Graphical abstract

A high‐speed embedded ink writing approach is proposed, which is powered by the development of a particle‐hydrogel interactive system to design suitable yield‐stress fluids. Using this approach, an anatomic‐size human kidney analog is printed in only 4.2 h at the speed ten times higher than the printing speed barrier of current methods, demonstrating the great potential for organ reconstruction.

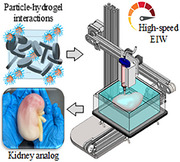

## 1 Introduction

Embedded ink writing (EIW), a new 3D printing strategy, has been rapidly developed in recent years. Because the geometrical complexity,^[^ ^1^, ^2^, ^3^ ^]^ range of printable feature size,^[^ ^2^, ^3^ ^]^ and ink material selection^[^ ^1^, ^2^ ^]^ in EIW comprehensively overwhelm those of other material extrusion‐based strategies, such as direct ink writing (DIW) and fused deposition modeling (FDM), it has been extensively utilized in diverse fields, like biomedical engineering,^[^ ^4^ ^]^ aerospace engineering,^[^ ^5^ ^]^ soft robotics,^[^ ^6^ ^]^ and wearable sensors,^[^ ^7^ ^]^ to name a few.

**Figure 1:**
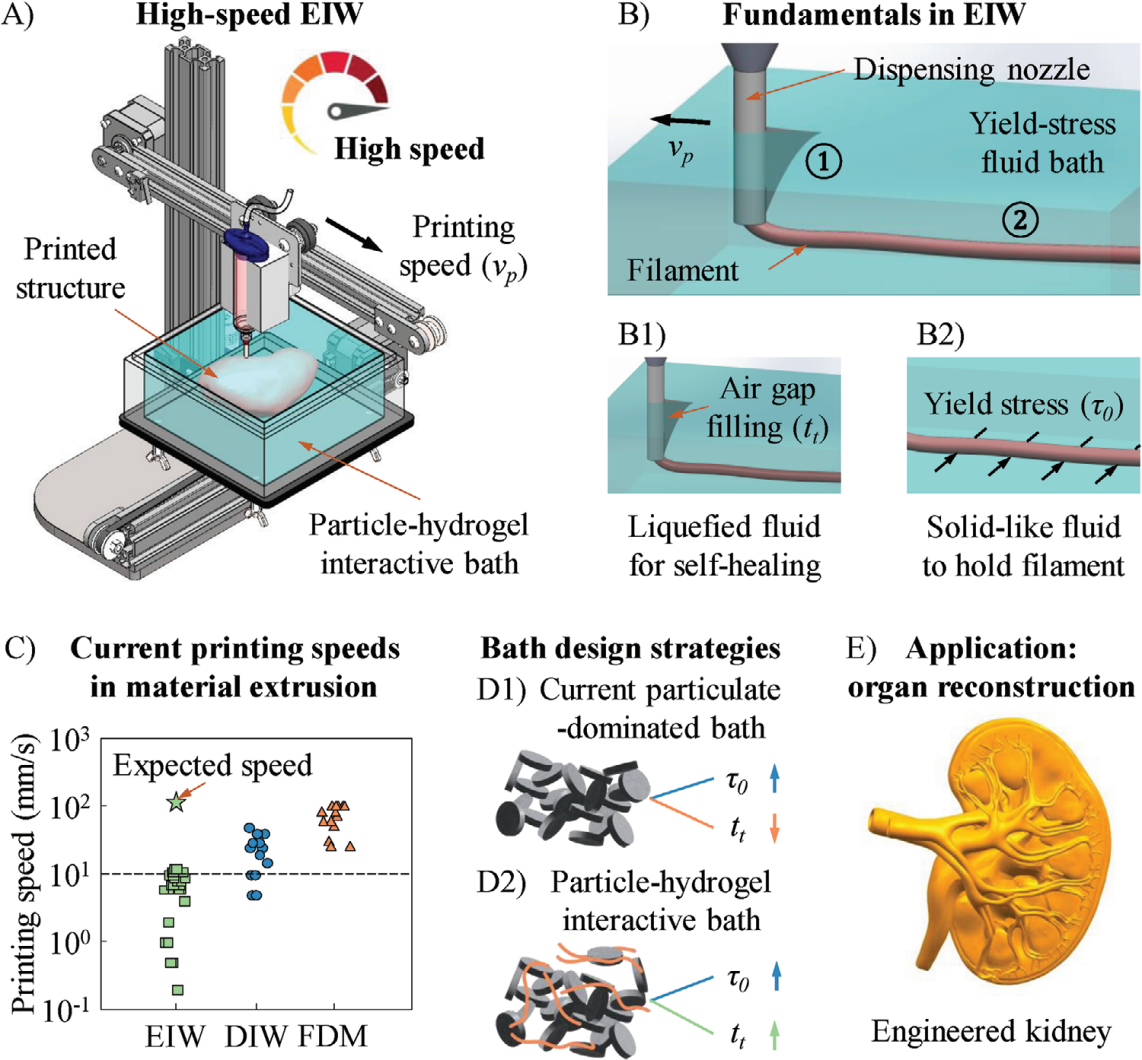
Fundamentals in EIW. A) Proposed high‐speed EIW approach. B) Two key physical phenomena in high‐speed EIW: B1) air gap filling affected by thixotropic response time (*t_t_*) and B2) filament holding determined by yield stress (*τ_0_*) of bath material. C) Summary of printing speed ranges of three material extrusion‐based printing strategies, including EIW, DIW, and FDM as well as the expected speed for high‐speed EIW. D) Bath design strategies: D1) current particulate‐dominated system and D2) proposed particle‐hydrogel interactive system. E) Representative biomedical application of high‐speed EIW approach: organ reconstruction with high fabrication efficiency.

The printing process in EIW is performed within a yield‐stress fluid with an inherent microstructure, which serves as the liquid bath to provide excellent “soft” supports (**Figure** **1**A). The disturbance and recovery of the microstructure enable the bath to repeatedly switch between liquid and solid‐like states at a macroscopic level.^[^ ^2^, ^8^, ^9^, ^10^ ^]^ Particularly, the transition from solid‐like to liquid at a higher shear stress allows a dispensing nozzle to move freely within the bath to deposit filaments from inks (Figure 1B). Meanwhile, the liquefied bath needs to rapidly fill the air crevasse behind the nozzle translation to entrap extruded filaments in situ (Figure 1B1). After the nozzle moves away, the decrease of localized shear stress drives the transition from liquid to solid‐like, giving the bath sufficient mechanical stiffness to stably hold the printed features before crosslinking (Figure 1B2). While the liquid bath greatly facilitates the construction of arbitrary 3D architectures, it also creates a unique and complex liquid‐liquid environment for filament formation in EIW, making uncross‐linked filaments more sensitive to the properties of the liquid bath,^[^ ^11^, ^12^ ^]^ printing conditions,^[^ ^13^, ^14^, ^15^ ^]^ and ink‐bath interactions.^[^ ^13^, ^16^, ^17^ ^]^ Thus, filament defects, such as over‐deposited filament,^[^ ^13^, ^18^ ^]^ non‐uniform filament,^[^ ^10^, ^19^ ^]^ shrinking filament,^[^ ^20^, ^21^ ^]^ sinking filament,^[^ ^2^, ^10^, ^20^ ^]^ etc., commonly occur especially when the printing speed is elevated. The characteristics of the liquid‐liquid environment constrain the current maximum printing speeds of EIW on the order of 10 mm s^−1^,^[^ ^11^, ^13^, ^20^, ^22^, ^23^, ^24^, ^25^, ^26^, ^27^, ^28^, ^29^, ^30^ ^]^ much lower than that of DIW and FDM,^[^ ^31^, ^32^, ^33^, ^34^, ^35^, ^36^ ^]^ as summarized in Figure 1C. Therefore, propelling printing speeds in EIW becomes a major engineering challenge that, if solved, can significantly enhance the fabrication efficiency and greatly broaden the application scope of this 3D printing technique.

Currently, the mainstream strategy to develop yield‐stress baths is to disperse specific particles, e.g., nanoclay,^[^ ^1^, ^27^, ^29^ ^]^ Carbopol,^[^ ^12^, ^37^, ^38^ ^]^ gelatin microparticles,^[^ ^25^, ^26^, ^39^ ^]^ and fumed silica,^[^ ^11^, ^28^ ^]^ into either an aqueous or an organic solvent to form a particulate‐dominated system. Due to attraction or repulsion between particles,^[^ ^2^ ^]^ an inherent microstructure can be formed in the solvent, giving the fluid a yield‐stress property. In these particulate‐dominated liquid baths, two key rheological parameters, yield stress (*τ_0_*) and thixotropic response time (*t_t_*), are both sensitive to particle concentration, but in opposite directions: the increase of particle concentration can enhance *τ_0_* while simultaneously shorten *t_t_*,^[^ ^2^, ^11^, ^28^ ^]^ as shown in Figure 1D1. The combination of rheological parameters in this case may lead to the formation of diverse filament deflects, especially at high printing speeds. For example, LeBlanc et al.^[^ ^19^ ^]^ observed permanent air gap behind a nozzle and non‐uniform filaments when *τ_0_* was too high and the bath material could not sustain a fluidized state with low viscosity for a sufficient time. Hua et al.^[^ ^20^ ^]^ printed in a matrix bath with a low *τ_0_* and a long *t_t_*. The formed filaments had a well‐defined cross‐sectional shape but shrank in the bath after printing. As a result, for high‐speed EIW, the bath must provide a high *τ_0_* to prevent filament over‐deposition, shrinkage, and sinking as well as possess a sufficiently long *t_t_* for filling the air gap behind the nozzle and ensuring the circular cross section of filaments. Based on these rheological expectations, we propose a particle‐hydrogel interactive system in this work, where the interactions between particle additive and hydrogel solvent not only enhances *τ_0_* greatly but also extends *t_t_* (Figure 1D2), resulting in a yield‐stress bath suitable for printing filaments with well‐defined geometries at high speeds (Figure 1A). This high‐speed EIW technique can be particularly effective in biomedical engineering to efficiently reconstruct anatomic‐size human tissues and organs (Figure 1E), which requires a minimized printing time to ensure a high cell viability after printing.^[^ ^40^ ^]^

## 2 Results and Discussion

### 2.1 Yield‐Stress Fluid Design Based on Particle‐Hydrogel Interactions

In this work, nanoclay, a classic yield‐stress particle additive,^[^ ^41^, ^42^ ^]^ is selected which has a high aspect ratio with diameter of ∼20–25 nm and thickness of 1 nm. When dispersed in an aqueous solvent, nanoclay platelets present positive and negative charges on the edge and faces, respectively, leading to the formation of a stable “house‐of‐cards” arrangement at electrostatic equilibrium.^[^ ^43^, ^44^ ^]^ The charge distribution makes nanoclay miscible with a variety of hydrogels via different interaction mechanisms.^[^ ^42^, ^45^ ^]^ To study the interactions between nanoclay particles and hydrogel solvents, three representative hydrogels are selected, including sodium alginate (NaAlg), polyethylene glycol diacrylate (PEGDA), and Pluronic F127. NaAlg is a negatively charged, ionically crosslinkable natural hydrogel that does not possess a given microstructure if uncross‐linked.^[^ ^46^ ^]^ PEGDA is a neutral, ultraviolet (UV) crosslinkable synthetic hydrogel without a given microstructure before crosslinking.^[^ ^47^ ^]^ Pluronic F127 is a thermosensitive triblock copolymer with two species, poly(ethylene oxide) (PEO) and poly(propylene oxide) (PPO). When the ambient temperature is below a critical micelle temperature (*T_cm_*), polymer chains of Pluronic F127 exist in water in an alternating linear PEO‐PPO‐PEO configuration. When the ambient temperature is above *T_cm_*, PEO‐PPO‐PEO species can form spherical micelles that consist of a hydrophobic PPO core surrounded by a hydrophilic PEO corona,^[^ ^48^ ^]^ giving Pluronic F127 an inherent microstructure. These three hydrogels are expected to represent the main categories of hydrogels in terms of chargeability and inherent microstructure. The obtained interaction mechanisms may guide the design of other nanocomposites composed of nanoclay and hydrogels (like gelatin, chitosan, hyaluronic acid, etc.) with desired rheological properties for diverse 3D printing applications.

After mixing nanoclay with three hydrogels at different formulas, the resulting nanocomposites present different states, as summarized in Figure S1 (Supporting Information). Particularly, the increase of alginate concentration leads to the yield‐stress transition of the nanoclay‐alginate nanocomposites from regular, enhanced, weakened, and to none. For example, when the nanoclay concentration is 6%, the maximum alginate concentration is ∼1% to make the nanocomposites retain a yield‐stress property. Above this threshold alginate concentration, the nanocomposites gradually lose yield‐stress behavior and become conventional pseudoplastic fluids with relatively high viscosities. In contrast, the nanoclay‐PEGDA nanocomposites always behave solid‐like under a low shear stress condition when the nanoclay and PEGDA concentrations vary in the ranges of 4–8% and 5–15%, respectively. However, the increase of nanoclay concentration enhances the yield‐stress property of the nanocomposite. The nanoclay‐Pluronic F127 nanocomposites face more complicated phase transitions. At a given nanoclay concentration (e.g., 2%), the nanocomposites are yield‐stress suspensions at lower concentrations of Pluronic F127 (e.g., 0.25%). When the Pluronic F127 concentration changes between 1 and 10%, the nanocomposites become viscous fluids. With a Pluronic F127 concentration above 20%, the nanocomposites regain a yield‐stress property and behave solid‐like again under the low shear stress condition. These experimental observations validate that different particle‐hydrogel interactions occur within the prepared nanocomposites, making them demonstrate various yield‐stress statuses.

### 2.2 Modeling Interactions between Nanoclay and Alginate

**Figure 2:**
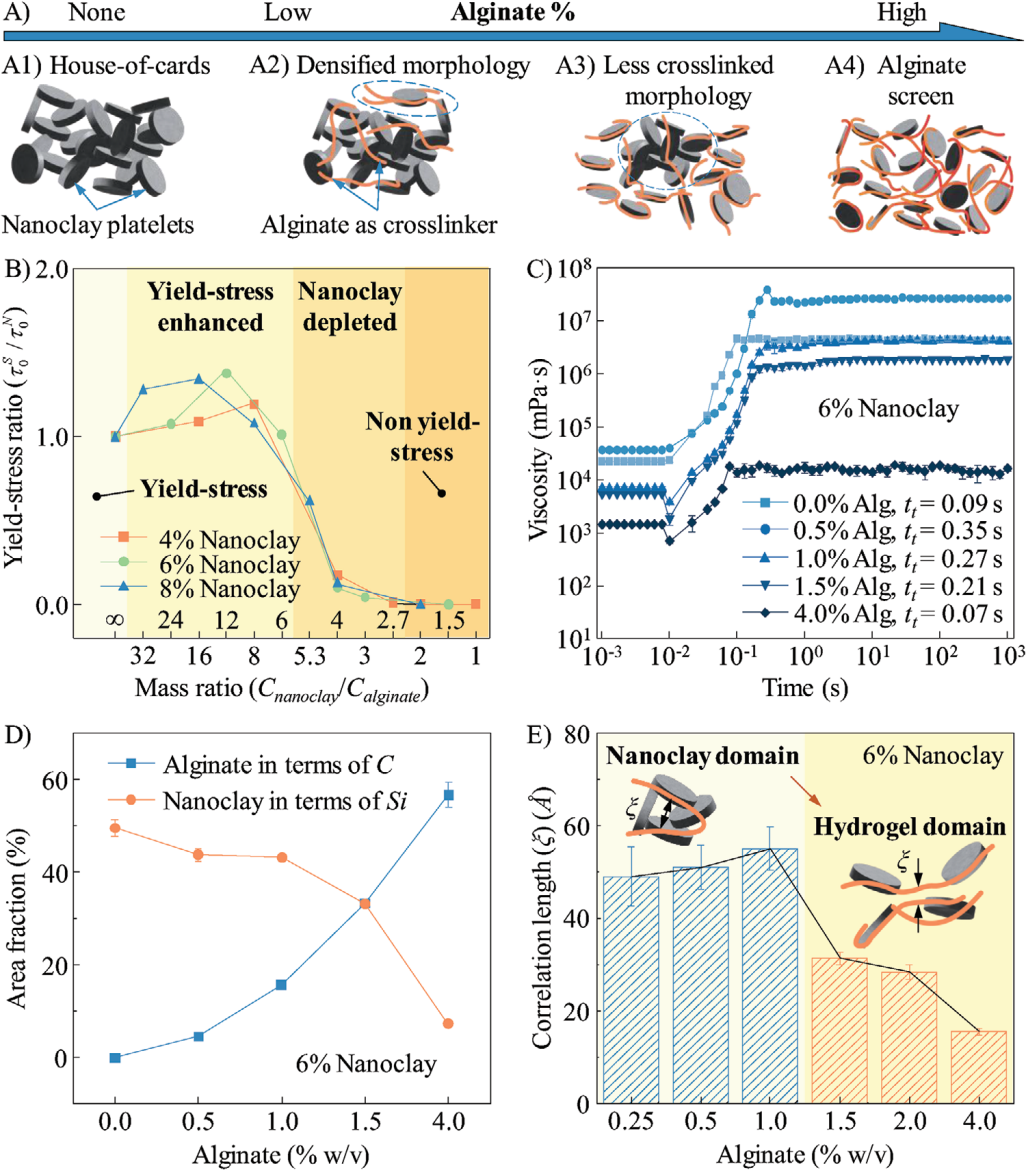
Interactions in the nanoclay‐alginate system. A) Hypothesized interaction models as affected by the formula of nanoclay‐alginate nanocomposites: A1) “house‐of‐cards” microstructure, A2) densified “house‐of‐cards”, A3) less crosslinked “house‐of‐cards”, and A4) collapsed “house‐of‐cards” with the increasing alginate concentration in the nanocomposites. Macroscale validation: B) yield‐stress ratio as a function of composition mass ratio to demonstrate four rheology regimes in the nanocomposites; and C) thixotropic response time of the nanocomposites with different concentrations of alginate. D) Microscale characterization via SEM and EDS: area fraction of silicon and carbon atoms as a function of alginate concentration. E) Nanoscale characterization via SANS: correlation length in the nanocomposites as a function of alginate concentration.

We propose the models in **Figure** **2**A to explain the potential interactions between nanoclay and alginate in the nanocomposites. For a pure nanoclay suspension without alginate, nanoclay platelets exist in the aqueous solvent in the form of a “house‐of‐cards” microstructure (Figure 2A1). A critical shear stress is needed to collapse this microstructure, triggering the transition from solid‐like to liquid, which is defined as the yield stress of the nanoclay suspension ($\tau_{0}^{N}$). When alginate is added at a low concentration, hydrogel polymer chains intersperse through the “house‐of‐cards” arrangement and absorb to the edges of nanoclay platelets via electrostatic interactions. Thus, polymer chains function as physical crosslinkers to shorten the distance between nanoclay platelets and densify the resulting microstructure within the nanocomposite (Figure 2A2), leading to enhanced yield‐stress behavior. When more alginate is added to the nanocomposite, hydrogel polymer chains start to snatch nanoclay platelets, causing the depletion of the “house‐of‐cards” arrangement and deterioration of yield‐stress behavior (Figure 2A3). When the alginate concentration is higher than a threshold value, nanoclay platelets prioritize the absorption on freely movable alginate polymer chains, resulting in the collapse of the “house‐of‐cards” arrangement (Figure 2A4). Thus, the nanocomposite switches from a yield‐stress fluid to a pseudoplastic liquid.

To validate the proposed interaction models at the macroscale, we measure the rheological properties of the nanoclay‐alginate nanocomposites. Their yield stress ($\tau_{0}^{S}$) values are illustrated in Figure S2 (Supporting Information). Specifically, for the nanocomposites with 6% nanoclay, when the alginate concentration increases from 0 to 0.5%, the yield stress increases from 173.4 to 242.7 Pa. However, when the alginate concentration keeps increasing from 1 to 2%, the yield stress decreases greatly from 174.8 to 7.8 Pa. The nanocomposite loses its yield stress when the alginate concentration exceeds 4%. Similar yield‐stress variation also occurs to the nanocomposites with 4% and 8% alginate. To better demonstrate the phase change in the nanoclay‐alginate nanocomposites, two dimensionless numbers are defined, including 1) mass ratio which is the ratio of nanoclay concentration (*C_nanoclay_*) to alginate concentration (*C_alginate_*) to standardize two compositions in the nanocomposites and 2) yield‐stress ratio which is the ratio of $\tau_{0}^{S}$ to $\tau_{0}^{N}$ for normalizing the rheological variation. Accordingly, the phase diagram is summarized in Figure 2B. For pure nanoclay, the mass ratio is infinite, and the suspension falls into the regular yield‐stress regime. When the mass ratio is at a relatively high level (e.g., from 6 to 32), the nanocomposites step into the yield‐stress enhanced regime with $\tau_{0}^{S}$ higher than $\tau_{0}^{N}$. With the decrease of mass ratio, the nanocomposites move into the nanoclay depleted regime and $\tau_{0}^{S}$ starts to decrease greatly. When the mass ratio is below a threshold value (e.g., 2 in this work), the nanocomposites do not have the yield‐stress behavior any more.

The change of alginate concentration also affects *t_t_*, as illustrated in Figure 2C. For pure 6% nanoclay, the measured thixotropic response time is only 0.09 s, which means the nanoclay suspension can rapidly restore its “house‐of‐cards” arrangement. However, with the increase of alginate concentration, *t_t_* extends to a maximum of 0.35 s when the nanocomposite's yield stress value reaches its maximum and then decreases to 0.07 s when the nanocomposite has no yield stress. These results validate our hypothesis that replacing the aqueous solvent with the hydrogel solvent enables the resulting nanocomposites to present the desired rheology potentially suitable for high‐speed EIW.

To prove the proposed interaction models at the microscale, we conduct scanning electron microscopy (SEM)/energy dispersive X‐ray spectroscopy (EDS) and small‐angle neutron scattering (SANS) to the nanoclay‐alginate nanocomposites. Their SEM images and EDS results are illustrated in Figure S3 (Supporting Information). Because silicon and carbon atoms only exist in nanoclay and alginate, respectively, we use the distributions of these atoms to demonstrate the interactions between nanoclay and alginate in each nanocomposite. As shown in Figure S3 (Supporting Information), with the increase of alginate concentration, more carbon atoms are present in the nanocomposite. When alginate is at a lower concentration (e.g., 0.5%), silicon atoms occupy more space in the nanocomposite with an area fraction of 43.7 ± 1.4%, much higher than the area fraction of carbon atoms (i.e., 4.6 ± 0.4%) in Figure 2D, indicating that nanoclay dominates the formation of the microstructure in the nanocomposite. As seen from Figure S3A (Supporting Information), most of the carbon atoms approach the silicon atoms, which demonstrates that hydrogel polymer chains attach to nanoclay platelets and serve as physical crosslinkers in the system. When the alginate concentration is added at 1%, the silicon area fraction changes negligibly but the carbon area fraction increases greatly to 15.6 ± 0.2%. In Figure S3B (Supporting Information), more carbon atoms are present and randomly distributed in the nanocomposite, showing that hydrogel polymer chains detach from nanoclay platelets. As the alginate concentration increases to a higher range (e.g., 4%), silicon atoms tend to cluster around carbon atoms (Figure S3D, Supporting Information). The carbon area fraction increases significantly, while the silicon area fraction decreases sharply to ∼7.2 ± 0.4%. This change may be attributed to mutual occlusion in the nanoclay clusters within the system. In this case, hydrogel polymer chains dominate the microstructure of nanocomposite and nanoclay's “house‐of‐cards” arrangement cannot exist.

To further support the explanations, we characterize the microstructures of different nanoclay‐alginate nanocomposites via SANS, as shown in Figure S4 (Supporting Information). In a nanoclay‐hydrogel system, correlation length (*ξ*), equivalent to persistence length, is commonly used to describe the polymer stiffness, which is defined as the distance over which the bonds are correlated along the polymer chain.^[^ ^49^, ^50^, ^51^ ^]^ Herein, the cylinder model^[^ ^52^ ^]^ (Equations (1) and (2)) is used first to obtain the form factor of the disc‐shaped nanoclay platelets and achieve the dimensions of the nanoclay platelets in the pure nanoclay suspensions. Then, the combination of the correlation length model^[^ ^53^ ^]^ and cylinder model is developed to quantify the *ξ* values as shown in Equations (3) and (4) as follows:

(1) $$I_{cylinder}\left(Q\right)=\frac{scale_{\mathrm{particle}}}{v_{P}}\cdot \int_{0}^{\pi /2}{F_{cylinder}}^{2}\left(Q,\alpha \right)\cdot \sin\alpha d\alpha +Background$$

(2) $$F_{cylinder}\left(Q,\alpha \right)=2\left(\rho_{p}-\rho_{s}\right)v_{p}\frac{\sin(\frac{1}{2}QL\cos\alpha )}{\frac{1}{2}QL\cos\alpha }\frac{J_{1}\left(QR\sin\alpha \right)}{QR\sin\alpha }$$

(3) $$I_{nanoclay\_polymer}\left(Q\right)=I_{correlation\_length}\left(Q\right)+I_{cylinder}\left(Q\right)+Background$$

(4) $$I_{correlation\_length}\left(Q\right)=\frac{A}{Q^{n}}+\frac{C}{1+{\left(Q\xi \right)}^{m}}+Background$$

where *scale_particle_* is the volume fraction of nanoclay platelets, *v_P_* is the volume of a nanoclay platelet, *ρ_p_* is the scattering length density of nanoclay, *ρ_s_* is the scattering length density of hydrogel solvent, *L* and *R* are the thickness and radius of nanoclay platelet in the “house‐of‐cards” arrangement, respectively, *A* is the Porod scaling factor, *C* is the Lorentzian scaling factor, *n* is the Porod exponent and *m* is the Lorentzian exponent, *Background* is the scattering contribution from an incoherent background*, α* is the angle between the axis of a nanoclay platelet and the scattering vector, *Q* is the scattering vector, and *J_1_* is the first‐order spherical Bessel function. The parameters for fitting the SANS data are summarized in Table S1 (Supporting Information). As shown in Figure 2E, two distinct regimes are divided in terms of correlation length: one is the particle phase domain and the other is the hydrogel phase domain. In the former, nanoclay platelets form the “house‐of‐cards” arrangement and alginate polymer chains absorb on the platelets’ edges. Therefore, *ξ* represents the distance between adjacent nanoclay platelets, which varies slightly at the alginate concentrations of 0.25 and 0.5%. When the concentration increases to 1%, *ξ* increases from 49.01 to 55.10 *Å*, illustrating that there is a looser microstructure with the nanocomposite. When the alginate concentration is above 1.5%, the microstructure switches to the hydrogel phase domain, where *ξ* is governed by the distance between alginate polymer chains instead of distance between nanoclay platelets. Thus, *ξ* is much lower in this domain and continuously decreases as the alginate concentration increases. The SANS data is consistent with the measured rheological properties of the nanoclay‐alginate nanocomposites in Figure 2B,C, which validates the proposed interaction models in Figure 2A.

### 2.3 Modeling Interactions Between Nanoclay and PEGDA

**Figure 3:**
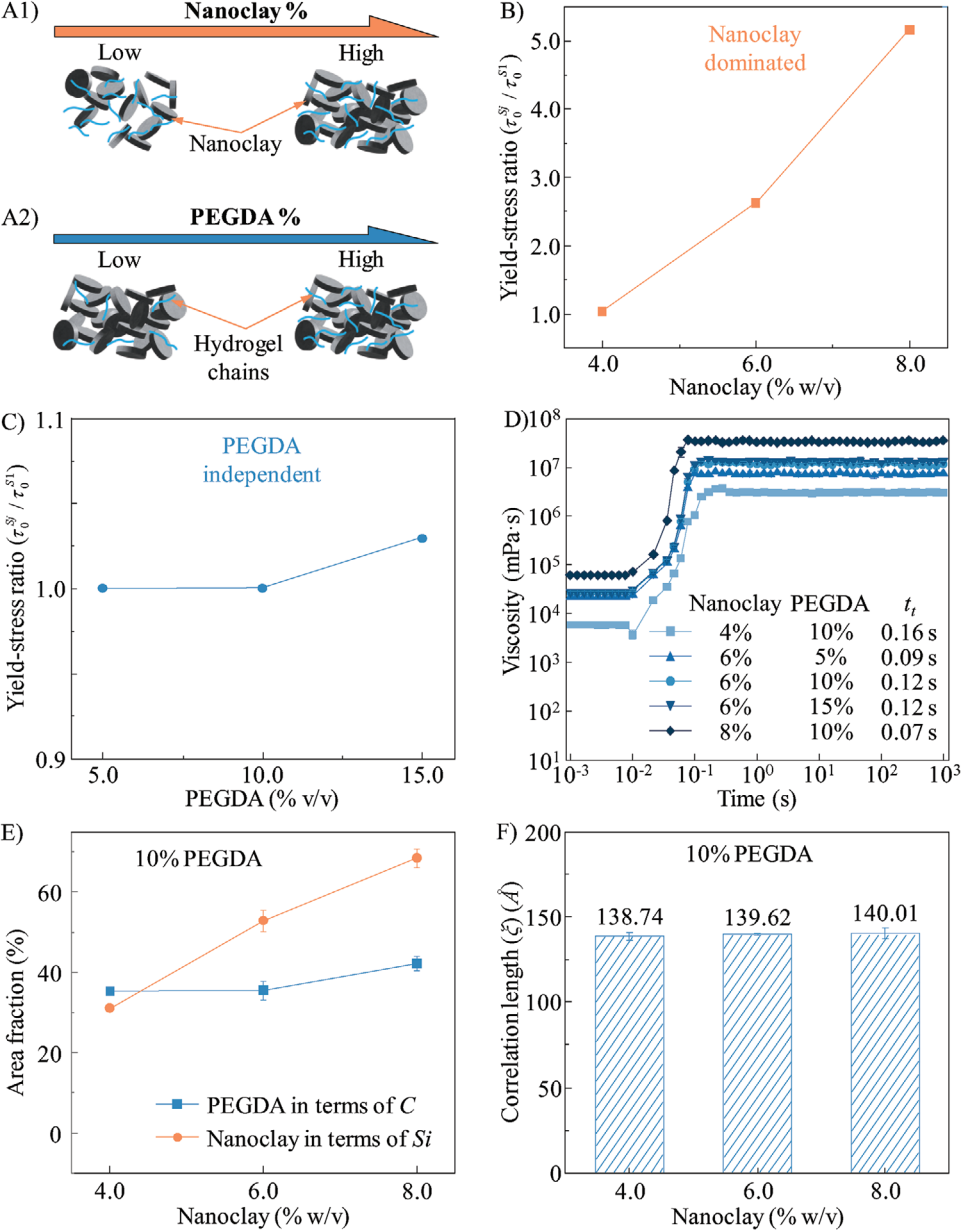
Interactions in the nanoclay‐PEGDA system. A) Hypothesized interaction models in the nanoclay‐PEGDA nanocomposites: “house‐of‐cards” microstructure A1) affected by nanoclay concentration and A2) independent on PEGDA concentration. Macroscale validation: yield‐stress ratio of the nanocomposites B) affected by nanoclay and C) independent on PEGDA; and D) thixotropic response time of the nanocomposites with different formulas. E) Microscale characterization via SEM and EDS: area fraction of silicon and carbon atoms as a function of nanoclay concentration. F) Nanoscale characterization via SANS: correlation length in the nanocomposites as a function of nanoclay concentration.

We propose the models in **Figure** **3**A to predict the potential interactions between nanoclay and PEGDA. Because PEGDA polymer chains are neutral, they can intersperse through the “house‐of‐cards” arrangement of nanoclay with weak interactions (such as ionic, dipole, and polymer entanglements), which enables the formed microstructure to be dominated by the nanoclay concentration (Figure 3A1) and be independent on the PEGDA concentration (Figure 3A2). These interaction models are supported by the rheological measurements. The $\tau_{0}^{S}$ values of different nanocomposites are illustrated in Figure S5 (Supporting Information). Moreover, the yield‐stress ratios as a function of nanoclay concentration and PEGDA concentration are summarized in Figure 3B,C, respectively. At the given PEGDA concentration (i.e., 10%), the addition of more nanoclay platelets greatly increases $\tau_{0}^{S}$. In contrast, at the same nanoclay concentration (i.e., 6%), the nanocomposites possess similar $\tau_{0}^{S}$ values when changing the concentration of PEGDA. The same phenomenon is also observed in the thixotropic response time measurements, as shown in Figure 3D. When the nanoclay concentration is 6%, the increase of PEGDA concentration from 5 to 15% only results in a slight variation of *t_t_* between 0.09 and 0.12 s. In contrast, when the nanoclay concentration increases from 4 to 8%, *t_t_* decreases significantly from 0.16 to 0.07 s. Thus, the rheological properties are mainly controlled by nanoclay concentration, similar to the method of tuning rheology in the conventional particulate‐dominated system. As a result, such nanocomposites may not be useful as yield‐stress fluids for high‐speed EIW.

The SEM images of different nanoclay‐PEGDA nanocomposites are illustrated in Figure S6 (Supporting Information). The element distributions are shown in Figure S7 (Supporting Information), in which silicon and carbon atoms are marked in blue and red colors to represent nanoclay and PEGDA, respectively. It is observed that silicon atoms interpenetrate with carbon atoms and no pronounced atom clusters are observed. The area fractions of the nanocomposites with 10% PEGDA and 6% nanoclay are calculated and illustrated in Figure 3E and Figure S8 (Supporting Information), respectively. When the PEGDA concentration is constant, the increase of nanoclay concentration only increases the area fraction of silicon atoms and has negligible effects on the area fraction of carbon atoms. The similar phenomenon is also observed when the nanoclay concentration is constant (Figure S8, Supporting Information). These findings demonstrate that nanoclay and PEGDA have negligible interactions when mixing together.

The SANS data of different nanoclay‐PEGDA nanocomposites (Figure S9, Supporting Information) also supports this hypothesis. The parameters for fitting the SANS data are summarized in Table S2 (Supporting Information). Using Equations (3) and (4), *ξ* value of each nanocomposite is calculated and summarized in Figure 3F and Figure S10 (Supporting Information). When the nanoclay concentration increases from 4 to 8%, *ξ* of the nanocomposites with 10% PEGDA slightly changes between 138.7 and 140.0 *Å*, which is because nanoclay platelets do not interact with PEGDA polymer chains. Thus, *ξ* mainly depends on the PEGDA concentration. In contrast, when the PEGDA concentration increases from 5 to 15%, *ξ* of the nanocomposites with 6% nanoclay decreases from 141.6 to 137.4 *Å* (Figure S10, Supporting Information) because the existence of more PEGDA polymer chains shortens the inter‐chain distance.

### 2.4 Modeling Interactions Between Nanoclay and Pluronic F127

**Figure 4:**
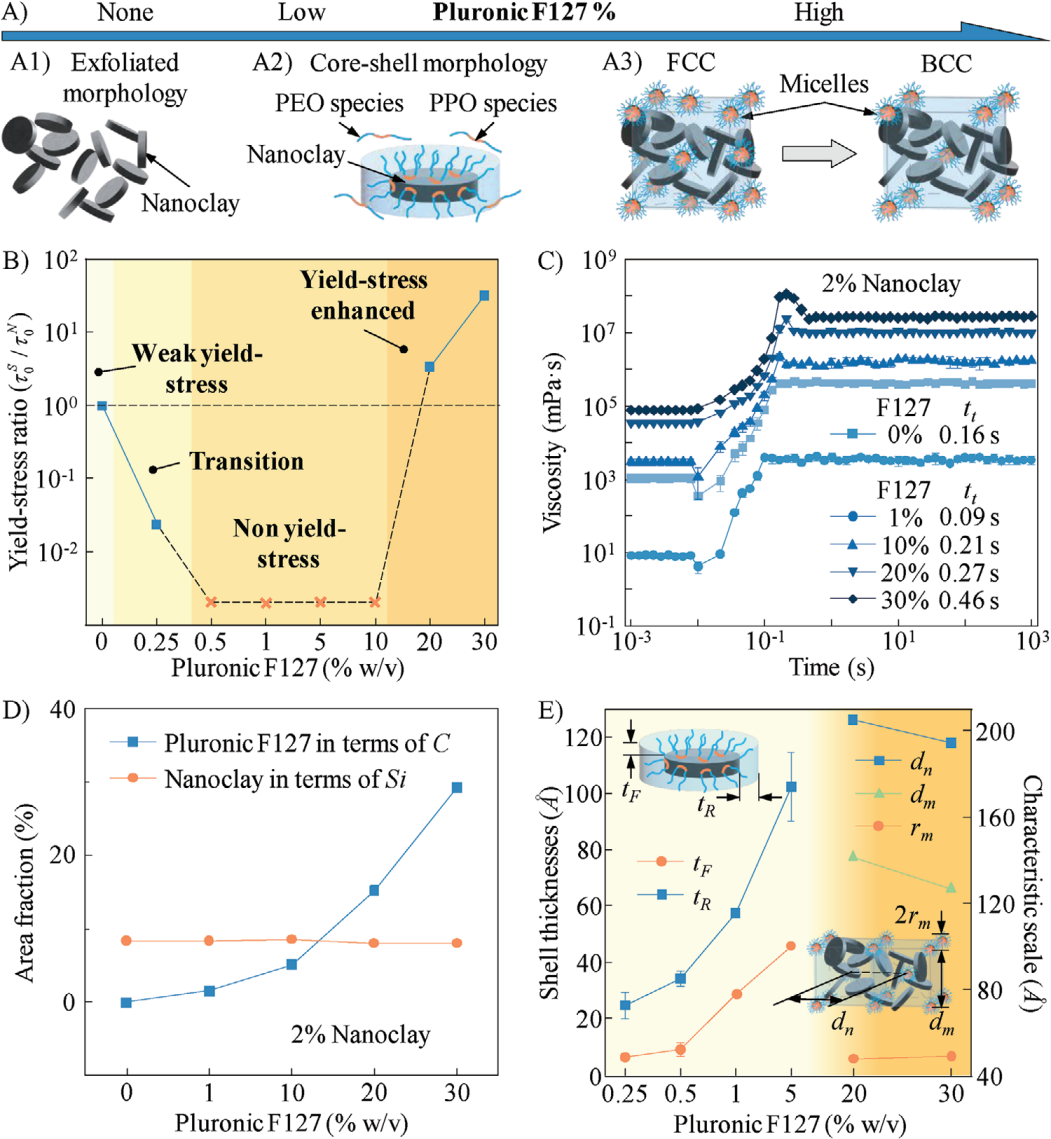
Interactions in the nanoclay‐Pluronic F127 system. A) Hypothesized interaction models as affected by the formula of nanoclay‐alginate nanocomposites: A1) exfoliated “house‐of‐cards” microstructure, A2) core‐shell morphology, and A3) FCC and BCC dual microstructures between nanoclay and Pluronic F127 micelles with the increasing Pluronic F127 concentration in the nanocomposites. Macroscale validation: B) yield‐stress ratio as a function of Pluronic F127 concentration to demonstrate four rheology regimes in the nanocomposites; and C) thixotropic response time of the nanocomposites with different Pluronic F127 concentrations. D) Microscale characterization via SEM and EDS: area fraction of silicon and carbon atoms as a function of Pluronic F127 concentration. E) Nanoscale characterization via SANS: correlation length in the nanocomposites as a function of Pluronic F127 concentration.

The proposed interaction models in the nanoclay‐Pluronic F127 nanocomposites are illustrated in **Figure** **4**A. Before adding Pluronic F127, nanoclay platelets show an exfoliated morphology (Figure 4A1) at a concentration of 2%.^[^ ^54^ ^]^ After mixing, the microstructure of Pluronic F127 is concentration dependent.^[^ ^55^, ^56^ ^]^ At a lower concentration, polymer chains of Pluronic F127 exist in the nanocomposite in the PEO‐PPO‐PEO configuration. Thus, hydrophobic PPO segments in polymer chains absorb on nanoclay platelets which are also hydrophobic,^[^ ^57^ ^]^ leading to the formation of a nanoclay platelet core surrounded by a shell from multiple Pluronic F127 polymer chains, as shown in Figure 4A2. This core‐shell morphology isolates the electrostatic interactions between adjacent nanoclay platelets, inhibiting the formation of the “house‐of‐cards” arrangement. Thus, the nanocomposite switches from a low yield‐stress fluid to a pseudoplastic liquid. With the increase of Pluronic F127 concentration (e.g., 20 or 30%), PEO‐PPO‐PEO species form spherical micelles, which not only release nanoclay platelets from encapsulation but also form a jammed microstructure of micelles with face‐centered cubic (FCC) or body‐centered cubic (BCC) symmetry.^[^ ^58^ ^]^ As a result, the recovered “house‐of‐card” arrangement from nanoclay integrates with the jammed microstructure from Pluronic F127, enabling the nanocomposite to possess an interactive dual microstructure, as shown in Figure 4A3. To collapse this dual microstructure, a higher shear stress is required which elevates the yield stress value. In addition, when the shear stress is removed, it takes a longer time to let the nanocomposite restore its dual microstructure, which extends the thixotropic response time.

The rheological properties are characterized to support our proposed interaction models. Particularly, the $\tau_{0}^{S}$ values of different nanoclay‐Pluronic F127 nanocomposites are measured in Figure S11 (Supporting Information) and the corresponding phase diagram is summarized in Figure 4B. In addition, the *t_t_* values of different nanocomposites are quantified in Figure 4C. It is observed that pure 2% nanoclay presents a low yield stress of 13.5 Pa and a relatively long thixotropic response time of 0.16 s. When Pluronic F127 is added at a lower concentration (e.g., 1%), the nanocomposite loses its yield‐stress property and behaves as a liquid in the non‐yield‐stress regime in Figure 4B. Increasing the concentration of Pluronic F127 above a threshold value enables the nanocomposite to move into the yield‐stress enhanced regime, regaining a much higher $\tau_{0}^{S}$ and a longer *t_t_*. For example, the nanocomposite with 30% Pluronic F127 has a yield stress of over 400 Pa and a thixotropic response time of 0.46 s, which perfectly fulfills our expectations of an ideal liquid bath for high‐speed EIW purpose. Such nanocomposites can stably hold the deposited filaments in situ even at high printing speeds as well as completely fill the crevasse behind the nozzle translation.

The SEM images of the nanoclay‐Pluronic F127 nanocomposites are shown in Figure S12 (Supporting Information), and the element distributions are illustrated in Figure S13 (Supporting Information). When Pluronic F127 is added at a lower concentration (e.g., 1%), carbon atoms aggregate with silicon atoms, which indicates the formation of core‐shell morphology (Figure 4A2). When the concentration of Pluronic F127 exceeds a threshold value (e.g., 20%), carbon atoms start clustering, demonstrating the formation of micelles. Meanwhile, silicon atoms have a constant area fraction over the increase of Pluronic F127 concentration (Figure 4D) and present similar element distributions when the Pluronic F127 concentration varies at 0 and 30%, which proves that nanoclay platelets regain the “house‐of‐cards” arrangement when micelles generate in the nanocomposites.

The characteristic dimensions of the nanoclay‐Pluronic F127 nanocomposites are calculated based on the SANS data (Figure S14, Supporting Information) using the following equations:^[^ ^57^, ^58^, ^59^ ^]^

(5) $$I_{core\_shell}\left(Q\right)=\frac{scale_{particle}}{v_{t}}\int_{0}^{\pi /2}{F_{core\_shell}}^{2}\left(Q,\alpha \right)\cdot \sin\alpha d\alpha +Background$$

(6) $$\begin{array}{ccc} F_{core\_shell}\left(Q,\alpha \right) & = & v_{t}\left(\rho_{l}-\rho_{s}\right)\frac{\sin\left[Q\left(L+2t_{F}\right)\left(\cos\alpha \right)/2\right]}{Q\left(L+2t_{F}\right)\left(\cos\alpha \right)/2}\frac{2J_{1}\left[Q(R+2t_{R})\sin\alpha \right]}{Q(R+2t_{R})\sin\alpha }\\ & & +v_{p}\left(\rho_{p}-\rho_{l}\right)\frac{\sin\left[QL(\cos\alpha )/2\right]}{QL(\cos\alpha )/2}\frac{2J_{1}\left[QR\sin\alpha \right]}{QR\sin\alpha } \end{array}$$

(7) $$I\left(Q\right)=\frac{scale_{m}}{V_{m}}V_{lattice}P_{sphere}\left(Q\right)Z_{paracrystalline}\left(Q\right)+Background$$

(8) $$V_{lattice\_FCC}=\frac{8\pi {r_{m}}^{3}}{3\sqrt{2}{d_{n}}^{3}}$$

(9) $$V_{lattice\_BCC}=\frac{\sqrt{3}\pi {r_{m}}^{3}}{{d_{n}}^{3}}$$

where *v_t_* is the total volume of a core‐shell unit, *ρ*_l_ is the scattering length density of the shell, *t*_F_ and *t*_R_ are the shell face thickness and shell rim thickness, respectively, *scale*_m_ is the volume fraction of Pluronic F127 phase, *V*_m_ is the volume of a Pluronic F127 spherical micelle, and *V*_lattice_ is the volume correction for the crystal structure. Additionally, *r_m_* is the micelle radius (micelle diameter *d_m_* = 2*r_m_*) and *d*_n_ is the nearest‐neighbor distance. *P*_sphere_(*Q*) and *Z*_paracrystalline_ (*Q*) are the form factor of the normalized spheres and the paracrystalline structure factor for BCC or FCC structures, respectively.^[^ ^58^, ^59^ ^]^ The parameters for fitting the SANS data are summarized in Tables S3 and S4 (Supporting Information). Particularly, the systematic particle size (*S = 2R*) of pure nanoclay with different concentrations (2, 4, 6, and 8%) are calculated using Equation (1) and illustrated in Figure S15 (Supporting Information) with the parameters for fitting in Table S5 (Supporting Information). For 2% nanoclay, *S* is 264.24 *Å*, which is close to the diameter of each nanoclay platelet (∼250.0 *Å*), indicating that a loose “house‐of‐card” microstructure is formed. With the increase of nanoclay concentration, the “house‐of‐card” microstructure becomes denser, causing the decrease of the *S* value (Figure S15B, Supporting Information). When Pluronic F127 is added at lower concentrations (from 0.25 to 5%), Equations (5) and (6) are used to determine the characteristic shell thicknesses, including *t_F_* and *t_R_*, of the core‐shell morphologies, as shown in Figure 4E. The increase of Pluronic F127 enables more PEO‐PPO‐PEO species to attach to nanoclay platelets, leading to the increase of both *t*_F_ and *t*_R_. When Pluronic F127 is added at higher concentrations (e.g., 20 and 30%), three characteristic scales, including *r*_m_, *d*_n_, and *d*_m_, are calculated using Equations (7)–(9). As seen from Figure 4E, *r*_m_ remains constant in this concentration range, indicating that Pluronic F127 micelles are completely formed. However, both *d*_n_ and *d*_m_ decrease with the increase of Pluronic F127 concentration. Thus, a denser jammed microstructure is generated when Pluronic F127 reaches 30%. The microstructure evolution is consistent with the rheological property change, which validates the proposed interaction models in Figure 4A.

### 2.5 Filament Formation in Particle‐Hydrogel Interactive Yield‐Stress Fluids at High Speeds

**Figure 5:**
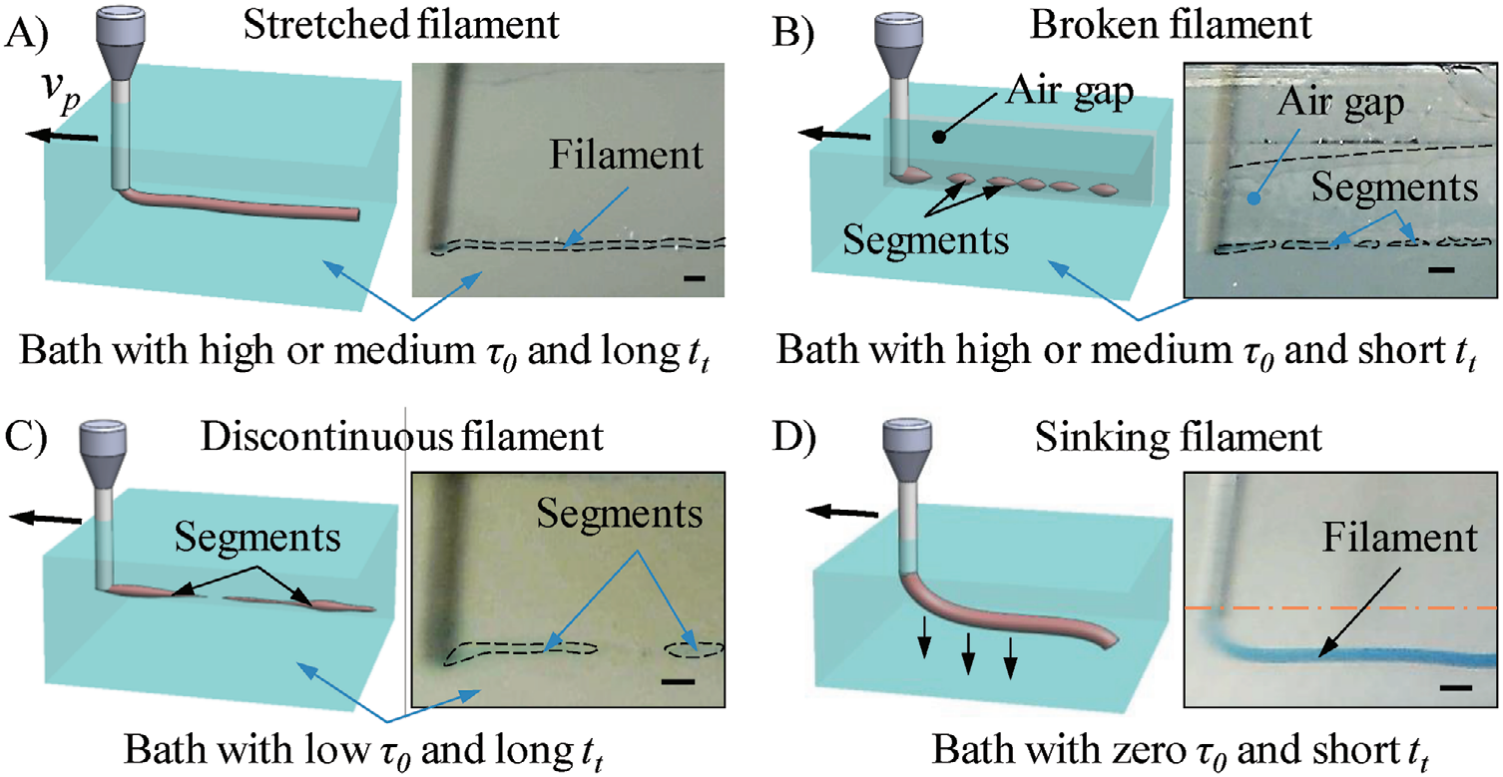
Filament formation in high‐speed EIW. A) Stretched filament in liquid bath with high or medium *τ_0_* and long *t*_t_. B) Broken filament in liquid bath with high or medium *τ_0_* and short *t*_t_. C) Discontinuous filament in liquid bath with low *τ_0_* and long *t*_t_. D) Sinking filament in liquid bath with zero *τ_0_* and short *t*_t_. Scale bars: 1.0 mm.

After understanding the interaction mechanisms of various nanoclay‐hydrogel nanocomposites, six representative nanocomposites are selected, each characterized by distinct *τ_0_* and *t_t_* falling within different ranges, as summarized in Table S6 (Supporting Information). A hydrogel ink consisting of alginate and PEGDA is printed into each nanocomposite at a speed of 110 mm s^−1^, which is ∼10 times higher than the conventional speeds employed in EIW.^[^ ^23^, ^24^, ^25^ ^]^ The formed filaments are illustrated in **Figure** **5**. As shown in Figure 5A and Movie S1 (Supporting Information), a well‐defined stretched filament with a diameter less than the nozzle inner diameter is generated in the nanocomposites with a high or medium *τ_0_* and a long *t_t_*. The relatively high *τ_0_* enables the nanocomposites to stably hold the filament in situ, effectively mitigating the adverse dragging effects due to fast nozzle movement during printing,^[^ ^17^, ^60^ ^]^ as well as counteracting the shrinkage of stretched filament after printing.^[^ ^13^, ^20^ ^]^ The long *t*_t_ allows the nanocomposites to remain in a liquid state long enough for the locally liquefied nanocomposites to completely fill the crevasse behind the nozzle translation.^[^ ^27^, ^30^ ^]^ Thus, it concludes that sufficient *τ_0_* and long *t*_t_ are required to facilitate filament formation at high printing speeds.

The afore‐discussed phenomena can be qualitatively predicted by two dimensionless ratios. The first ratio quantifies the relationship between the liquid bath's viscous stress (σvbath=K(vp/vpDNDN)n) and the ink's viscous stress ($\sigma_{v}^{ink}=\eta v_{ink}/D_{in}$) as $Fi=\sigma_{v}^{bath}/\sigma_{v}^{ink}$, where *K* and *n* are the consistency index and flow index in the Herschel‐Bulkley model, *v*_p_ and *v*_ink_ are the printing speed and ink speed, *D_N_* and *D_in_* are the nozzle outer and inner diameters, and *η* is the ink's viscosity. Because the thixotropic length scale of the liquid bath for EIW is close to the nozzle outer diameter as reported in some articles,^[^ ^11^, ^27^ ^]^ the bath's viscous stress can also be expressed as $\sigma_{v}^{bath}=K/t_{t}^{n}$, resulting in $Fi=\frac{KD_{in}}{t_{t}^{n}\eta v_{ink}}$, which can be applied to unveil the crevasse filling behind the nozzle translation. A lower *F_i_* indicates the crevasse prefers to be filled by the liquefied bath rather than being occupied by the up‐flow of the extruded ink, which requires the design of a liquid bath with longer *t*_t_. The second ratio involves the as‐deposited filament's elastic stress in relation to the liquid bath's *τ_0_* as $Ho=\frac{G\cdot (v_{p}-v_{ink})}{v_{ink}\tau_{0}}$ (where *G* is the ink's elastic modulus), which can be used to predict the holdability of liquid bath for high‐speed EIW. A lower *Ho* indicates that the liquid bath has a better capability of holding printed filaments stably in situ. As a result, *τ_0_* needs to be high when the printing speed is increased.

When high‐speed printing in the nanocomposites with relatively high *τ_0_* and short *t*_t_, the fast movement of the nozzle leads to a high shear stress, which induces the liquefaction of the nanocomposites around the nozzle. However, these localized nanocomposites rapidly revert to a solid‐like state and stop flowing when the nozzle moves away, leaving an air gap behind the nozzle, as shown in Figure 5B and Movie S2 (Supporting Information). This air gap creates an air‐liquid interface to the deposited filament, leading to breakage due to surface tension.^[^ ^24^, ^28^ ^]^ Thus, only segments are observed in the nanocomposites.

Figure 5C and Movie S3 (Supporting Information) illustrate a similar segmentation phenomenon in a nanocomposite characterized by a low *τ_0_* and a long *t*_t_, albeit with a different formation mechanism. At a high printing speed, the deposited filament is stretched due to the dragging effects of the nozzle, which tends to shrink during and/or after printing. Because of the low *τ_0_*, the nanocomposite cannot hold the filament in place, which shrinks non‐uniformly and releases the residual tensile stress, eventually resulting in the filament breakup. An extreme case is the filament printing within a non‐yield‐stress nanocomposite, as illustrated in Figure 5D and Movie S4 (Supporting Information). The extruded filament moves together with the nozzle during printing, which has a diameter close to the nozzle's inner diameter. After printing, the filament sinks to the bottom of the container because the nanocomposite cannot provide any mechanical support.

### 2.6 High‐Speed EIW of Anatomic‐Size Human Organ Analogs

After understanding the filament formation mechanisms, we print an anatomic‐size human kidney analog at 110 mm s^−1^. Herein, 2% nanoclay‐30% Pluronic F127 nanocomposite is selected to serve as the liquid bath not only because of its suitable rheological properties for high‐speed EIW, but also due to its thermal sensitivity, which makes this nanocomposite addable at 4 °C during the fabrication process.^[^ ^30^, ^61^, ^62^ ^]^ Thus, it is technically feasible to use a short nozzle to print the human kidney analog, effectively minimizing extrusion‐induced cell damage if living cells are included. The same hydrogel ink for filament printing is used to print the kidney analog.

**Figure 6:**
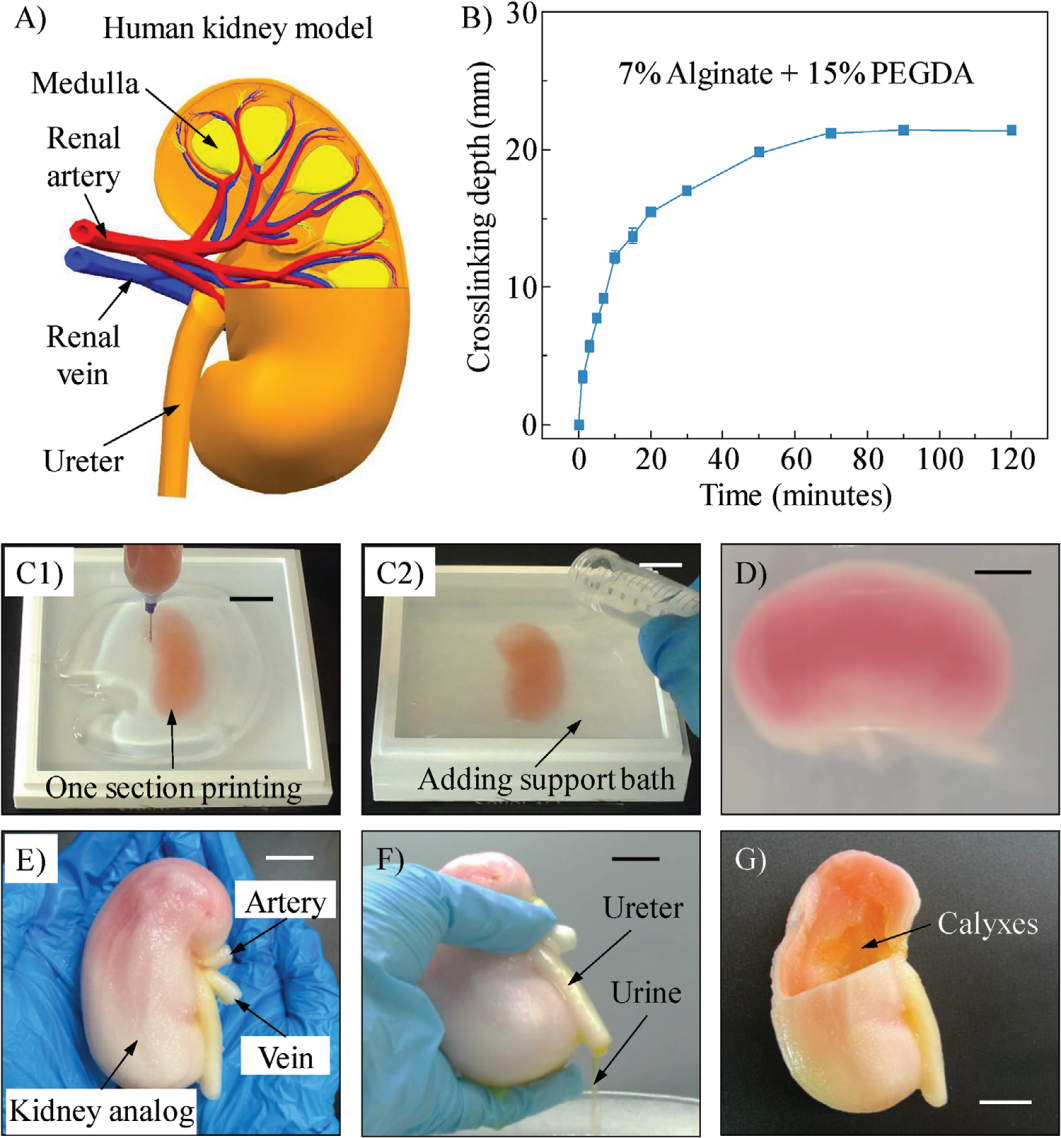
High‐speed EIW of anatomic‐size human kidney analog. A) Anatomic model of human kidney. B) Crosslinking depth of alginate‐PEGDA ink as a function of exposure time. C) Printing processes: C1) printing one section of the kidney within the 2% nanoclay‐30% Pluronic F127 nanocomposite and C2) adding 4 °C nanocomposite to the stackable container. D) The kidney analog after crosslinking. E) Anatomic‐size human kidney analog after post‐treatments. F) Functional demonstration of the printed kidney analog. G) Inner structure of the kidney analog. Scale bars: 2.0 cm.

The anatomic‐size human kidney model has the overall sizes of ∼99 × 43 × 37 mm^3^, as shown in **Figure** **6**A. Before printing, we characterize the crosslinking depth of the hydrogel ink to ensure the entire as‐printed kidney analog can be photo‐cross‐linked under UV radiation. The effects of exposure time on the cross‐linking depth are illustrated in Figure 6B. A maximum crosslinking depth of ∼21 mm is achieved after exposing to the UV light for 70 min, which is consistent with our previous study.^[^ ^63^ ^]^ Given the thickness of the designed kidney model (37 mm), bidirectional UV radiation for 70 min on each side is applied to guarantee the full cross‐linking of PEGDA component through the kidney analog, which also determines the printing orientation. A 21‐gauge nozzle with a length of 25.4 mm is selected based on this orientation as well as the height of each piece of the container. The kidney model is divided into four sections accordingly and the printing trajectory of each section is created.

In the printing process, the nanocomposite is perfused at 4 °C into the first piece of the stackable container, at which the nanocomposite presents a sol state with good flowability. When the nanocomposite's temperature recovers to room temperature, the nozzle moves in the nanocomposite to print the first section of the kidney model layer by layer, as shown in Figure 6C1. After that, the nanocomposite at 4 °C is added into the container again (Figure 6C2). By repeating this ink printing‐nanocomposite adding cycle several times, the anatomic‐size human kidney analog is printed section by section. The total fabrication time is ∼4.2 h, including 2.7 h for kidney printing and 1.5 h for adding the nanocomposite and recovering its temperature, as shown in Movie S5 (Supporting Information). Note that, the 3D bioprinter used in this work allows for a maximum speed of ∼120 mm s^−1^. To protect the machine, we select 110 mm s^−1^ as the printing speed, which may not reach the speed limitation of the chosen nanoclay‐Pluronic F127 nanocomposite. Higher printing speeds will be tested in the future, which can further shorten the fabrication time for reconstructing full‐scale and anatomic‐level human organ analogs.

After printing, the kidney analog embedded in the nanocomposite is exposed to UV radiation to chemically crosslink the PEGDA component. Then, the ambient temperature is decreased to 4 °C for liquefying the nanocomposite. Thus, it is easy to harvest the kidney analog from the liquid bath to physically crosslink the alginate component in a calcium chloride solution, as shown in Figure 6D. These post‐treatments are recorded in Movie S6 (Supporting Information). The printed anatomic‐size kidney analog is illustrated in Figure 6E and Movie S7 (Supporting Information), which demonstrates the well‐defined overall architecture of the renal artery, vein, and ureter structures. The key dimensions of the kidney analog are measured in Figure S16 (Supporting Information). The relative errors in Table S7 (Supporting Information) are all less than 2%, validating the proposed high‐speed EIW is able to fabricate organ analogs with both high efficiency and high accuracy.

To test the functionality of the printed kidney analog, we perfuse DI water with yellow‐colored dye through the ureter into the kidney analog. After flipping over, DI water gradually drips down to mimic the process of urine flowing past the ureter, as demonstrated in Figure 6F and Movie S8 (Supporting Information). To further explore the inner anatomic structure, we remove one section of the kidney analog, as shown in Figure 6G. The calyxes are clearly observed (Movie S9, Supporting Information), which validates that the inner structure of a human organ analog can be duplicated by the high‐speed EIW process. Given its unique advantages over other 3D bioprinting techniques (as summarized in Table S8, Supporting Information), the proposed high‐speed EIW is expected to be a promising method for future organ reconstruction.

## 3 Conclusion

In this work, the interactions between nanoclay and hydrogels diversify the rheological behaviors of the resulting nanocomposites. Different interaction models are proposed and supported by macroscale rheological measurements and microstructure characterizations. Serving as liquid baths, different categories of filaments are generated within the nanocomposites because of their rheological variation. The nanocomposite with high *τ_0_* and long *t*_t_ can greatly propel the printing speed to be above 100 mm s^−1^, which is 10 times higher than the commonly used speeds in current EIW. Benefiting from this high speed, an anatomic‐size human kidney analog is successfully printed in 4.2 h, which demonstrates a well‐defined outer structure as well as detailed inner structures.

In future work, we will use the interaction models to design more nanocomposites, which enables even higher printing speeds. Also, numerous filaments will be printed in these nanocomposites at elevated speeds to establish a relationship among the rheological properties of liquid bath, ink material properties, and key printing parameters, which will provide a theoretical guideline for the rational design of liquid baths for the high‐speed EIW applications. In addition, 3D printers customized for high‐speed EIW will be developed, which not only move much faster than current printers but also possess the functionality to automatically add thermosensitive nanocomposites during printing. Thus, it is possible to further shorten the fabrication cycle in human organ reconstruction. Finally, we will use living cells to prepare cellular bioinks and investigate the feasibility of printing cell‐laden human organ analogs.

## 4 Experimental Section

### Preparation of Nanoclay‐Hydrogel Nanocomposites

Three categories of nanoclay‐hydrogel nanocomposites were prepared in this work. For the nanoclay‐alginate nanocomposites, sodium alginate (NaAlg) powder (NaC_6_H_7_O_6_, Sigma–Aldrich, Burlington, MA) was first dispersed into deionized (DI) water at room temperature (25 °C) at the concentrations of 0.0, 0.25, 0.5, 1.0, 1.5, 2.0, and 4.0% (w/v) and continuously mixed using an overhead stirrer (50006‐03, Cole Parmer, Vernon Hills, IL) at 800 rpm for 40 min to make the NaAlg solutions. Then, dry nanoclay powder (Laponite RD, Na_0.7_Si_8_Mg_5.5_Li_0.3_O_20_(OH)_4_, BYK Additives Inc., Gonzales, TX) was dispersed into the solutions at the concentrations of 4.0, 6.0, and 8.0% (w/v) and mixed using the overhead stirrer to prepare the nanocomposites. The stirring speed was 600 rpm for at least 60 min to ensure the thorough hydration of nanoclay. For the nanoclay‐PEGDA nanocomposites, DI water was first added into a PEGDA (Mn 700, Sigma–Aldrich, Burlington, MA) stock solution at room temperature to dilute it to the concentrations of 5.0, 10.0, and 15.0% (v/v), respectively. The overhead stirrer was used for mixing at the speed of 300 rpm for 5 min. Then, nanoclay powder was dispersed into each solution at the concentrations of 4.0, 6.0, and 8.0% (w/v) and mixed using the stirrer (speed: 600 rpm and time: 60 min) to prepare the nanocomposites. For the nanoclay‐Pluronic F127 nanocomposites, 2.0% (w/v) nanoclay suspension was prepared first by adding and mixing the appropriate amount of dry nanoclay powder with DI water at room temperature using the overhead stirrer. The stirring speed was 600 rpm for at least 40 min to ensure the thorough hydration of nanoclay. Then, Pluronic F127 powder (P2443, Sigma‐Aldrich, Burlington, MA) was added to the nanoclay suspension at the concentrations of 0.0, 0.25, 0.5, 1.0, 5.0, 10.0, 20.0, and 30.0% (w/v) and mixed at 4 °C using the overhead stirrer at 800 rpm until the clear and homogenous nanocomposites were obtained. After preparation, all nanocomposites were degassed using a centrifuge (Cole‐Parmer VS3400, Cole Parmer Instrument Company, Vernon Hills, IL) at 2500 rpm for 2 min to remove entrapped air bubbles. These bubble‐free nanocomposites were stored in dark and sealed containers and aged for one day before use. Specifically, to observe the status, the nanocomposites were loaded into the glass vials (10 ml, VWR vials, Radnor, PA) with given inclined angles and imaged by a digital camera (DC‐FZ80, Panasonic, Osaka, Japan).

### Characterization of Rheological Properties

A rheometer (MCR 92, Anton Paar, Ashland, VA) with a cone‐plate measuring system (cone angle: 1°, cone diameter: 50.0 mm, and cone‐to‐plate gap: 0.102 mm) was used to characterize the rheological properties of the prepared nanocomposites. The steady shear rate sweeps were conducted to measure the yield stress values, in which the shear rate was increased from 0.01 to 1000 s^−1^, and the shear stress was recorded. Based on the shear stress‐shear rate curve, the yield stress of each nanocomposite was calculated using the Herschel‐Bulkley model. The transient step shear rate sweeps were performed to assess the thixotropic response time by pre‐shearing the nanocomposites at a relatively high shear rate of 10 s^−1^ for 200 s and then decreasing the shear rate to a low value of 0.01 s^−1^. The viscosity variation was recorded at this low shear rate for 1000 s. All measurements were performed at room temperature.

### Scanning Electron Microscopy (SEM) Imaging and Energy Dispersive X‐Ray Spectroscopy (EDS) Analysis

The microstructures within the nanocomposites were characterized using the Zeiss Merlin SEM and Bruker's EDS system, respectively, at the Center for Nanophase Materials Sciences at the Oak Ridge National Laboratory. For SEM imaging, the nanocomposites were loaded to holey carbon‐coated copper grids, air‐dried, and then imaged at 10 KeV. EDS analysis was performed using a QUANTAX EDS system integrated with the SEM system mentioned above. Elemental composition and distribution were determined by acquiring EDS spectra and mapping data. To eliminate noises from uneven background and thickness variation, the images were flattened with an open‐source software Gwyddion (http://gwyddion.net/) with a false color gradient called “Spectral”.

### Small‐Angle Neutron Scattering (SANS) Measurements

The extended Q‐range (EQ)‐SANS diffractometer^[^ ^64^ ^]^ at the Spallation Neutron Source at the Oak Ridge National Laboratory was used to further characterize the microstructures within different nanocomposites. All nanocomposites were prepared per the aforementioned protocol by replacing DI water with deuterium oxide (D_2_O, 99.9 atom % D, Sigma–Aldrich, Burlington, MA). Two sample‐to‐detector distances (i.e., 2.5 and 4.0 m) were applied with two minimum wavelength settings (i.e., *λ_min_* = 2.5 and 10.0 Å), respectively, to investigate scattering wave vectors ranging from 0.005 to 0.6 Å^−1^. The choppers were operated at 60 Hz. All nanocomposite samples were loaded in 2.0 mm quartz cells and measured at room temperature. The obtained data sets were corrected by detector sensitivity and background scatterings and then converted into absolute scale intensities (cm^−1^) using a porous silica standard sample.^[^ ^65^ ^]^ The open‐source software SasView (https://www.sasview.org/) was utilized to analyze the measured data and determine the microstructures of different nanocomposites. Acquiring structural parameters for nanoclay‐alginate and nanoclay‐PEGDA nanocomposites involved two principal procedures. Initially, the cylinder model was applied to fit the scattering data from pure nanoclay with the required parameters. Then, the obtained values of systematic particle radius and particle thickness were incorporated into the customized nanoclay‐hydrogel fitting model to fit the scattering data of the nanocomposites. The scattering data for nanoclay‐Pluronic F127 nanocomposites in the non‐yield‐stress regime was fitted using a core‐shell model, and the data in the yield‐stress enhanced regime was fitted using the face‐centered cubic (FCC) and body‐centered cubic (BCC) models. Detailed information on the fitting parameters was summarized in the Supporting Information.

### Filament Printing

The hydrogel ink composed of NaAlg and PEGDA was used to print filaments to investigate filament formation within different liquid baths. For the ink preparation, DI water was first added to the PEGDA stock solution to dilute it to the concentration of 15% (v/v). The overhead stirrer was used to uniformly mix water and PEGDA at 300 rpm for 5 min at room temperature. Then, 0.1% (w/v) photoinitiator I2959 (2‐hydroxy‐4′‐(2‐hydroxyethoxy)−2‐methylpropiophenone, Sigma–Aldrich, Burlington, MA) was mixed with the PEGDA solution for 5 min. After that, NaAlg powder was dispersed into the solution at the concentration of 7.0% (w/v) and continuously mixed using the overhead stirrer at 800 rpm for 60 min. Finally, the prepared hydrogel ink was degassed in the centrifuge at 2500 rpm for 2 min and stored in a sealed container before use. For the filament printing, six nanocomposites with representative yield stresses and thixotropic response times were selected and served as the liquid baths, including 2.0% (w/v) nanoclay‐30.0% (w/v) Pluronic F127, 8.0% (w/v) nanoclay‐10.0% (v/v) PEGDA, 6.0% (w/v) nanoclay‐1.0% (w/v) NaAlg, 6.0% (w/v) nanoclay‐10.0% (v/v) PEGDA, 6.0% (w/v) nanoclay‐1.5% (w/v) NaAlg, and 2.0% (w/v) nanoclay‐1.0% (w/v) Pluronic F127. A 21‐gauge nozzle (EFD Nordson, Vilters, Switzerland) with an inner diameter of 0.51 mm and a length of 12.7 mm was used to deposit the filaments from the hydrogel ink at the dispensing pressure of 20 psi (∼138 kPa) and printing speed of 110.0 mm s^−1^. To improve the visibility, a blue dye (Nile blue A, Sigma‐Aldrich, Burlington, MA) was added to the hydrogel ink at 0.01% (w/v). A camera (120fps USB Camera, ELP, Shenzhen, China) was applied to record the filament formation process from the front view. After printing, a high‐precision measurement system (VERTEX 251UC, Micro‐Vu, Windsor, CA) was used to observe the filament morphology within the liquid baths from the top view. The dimensions in the images were measured using ImageJ (https://imagej.nih.gov/ij/).

### Characterization of Cross‐linking Depth

The cross‐linking depth of the NaAlg‐PEGDA ink was characterized using the following procedures. First, the ink was filled into a centrifuge tube (15 mL, Membrane Solutions, Auburn, WA), which was trimmed to ∼50 mm in height and enveloped by aluminum foil. Then, the tube with the ink was placed 1.5 cm below a UV curing system (OmniCure Series 2000, wavelength: 320–500 nm, Lumen Dynamics, Mississauga, Canada). After that, the exposure time was increased from 0 to 120 min. At each time interval (1, 3, 5, 7, 10, 15, 20, 30, 50, 70, 90, and 120 min), the uncross‐linked ink was exhausted from the bottom and the length of the cross‐linked section was measured as the crosslinking depth.

### High‐Speed EIW of Anatomic‐Size Human Kidney Analog

The same hydrogel ink was used to print an anatomic‐size human kidney analog within the 2.0% (w/v) nanoclay‐30.0% (w/v) Pluronic F127 nanocomposite. To improve the visibility, red food dye (McCormick & Co., INC., Hunt Valley, MD) was added to the ink at 0.1% (v/v). A homemade extrusion 3D printing system modified based on a fused deposition modeling (FDM) 3D printer (SOVOL 3D, Shenzhen Liandian Technology Co., Ltd., Shenzhen, China) was utilized to print the kidney analog at room temperature. The printing parameters were listed as follows: 21‐gauge nozzle with a length of 25.4 mm, pressure of 50 psi, printing speed of 110.0 mm s^−1^, and step distance of 0.5 mm. The kidney model was directly downloaded from https://www.3d66.com/ as an STL file, which had the overall dimensions of 98.65 × 43.47 × 36.88 mm^3^. The model was equally segmented into 4 sections with a height of 9.22 mm for each section. A stackable container was designed, which had four pieces, and each piece had an inner length of 120.0 mm, inner width of 120.0 mm, and height of 15.0 mm. Each piece of the container was printed using the FDM 3D printer (SOVOL 3D, Shenzhen Liandian Technology Co., Ltd., Shenzhen, China). After printing one section of the kidney analog, additional nanoclay‐Pluronic F127 nanocomposite at 4 °C was perfused into the newly amounted piece of the container. The subsequent section was printed in 30 min when the temperature of the nanocomposite reached room temperature. After four sections were printed, the kidney analog within the container was exposed to UV radiation provided by the UV curing system for 70 min and then was moved into the refrigerator (4 °C) for 30 min to liquefy the nanocomposite. Finally, the kidney analog was harvested from the liquid bath and submerged in a 2.0% (w/v) calcium chloride (C8106, Sigma–Aldrich, Burlington, MA) solution for 24 h for ionic cross‐linking. To demonstrate the functionality, a syringe (10 ml, Medicore, Nashville, TN) was used to manually perfuse yellow‐colored DI water into the cross‐linked kidney analog. In addition, a scalpel was applied to remove one section of the kidney to observe its inner architecture.

### Statistical Analysis

All quantitative values of measurement in the figures were reported as means ± standard deviation (SD) with *n* = 3 samples per group.

## Conflict of Interest

The authors declare no conflict of interest.

## Supporting information



Supporting Information

Supplemental Movie 1

Supplemental Movie 2

Supplemental Movie 3

Supplemental Movie 4

Supplemental Movie 5

Supplemental Movie 6

Supplemental Movie 7

Supplemental Movie 8

Supplemental Movie 9

## Data Availability

The data that support the findings of this study are available from the corresponding author upon reasonable request.
