# Supplementary material for: High‐Speed Embedded Ink Writing of Anatomic‐Size Organ Constructs
Source: Adv Sci (Weinh). 2025 Feb 11;12(13):2405980. doi: 10.1002/advs.202405980 (PMC11967790; doi:10.1002/advs.202405980)
Supplement: Supplementary file 1 — Supporting Information [file ADVS-12-2405980-s008.docx]

Supporting Information

High-Speed Embedded Ink Writing of Anatomic-Size Organ Constructs

Weijian Hua^1, †^, Cheng Zhang^1, 2, †^, Haoran Cui^1^, Kellen Mitchell^1^, Dale K. Hensley^3^, Jihua Chen^3^, Changwoo Do^4^, Lily Raymond^1^, Ryan Coulter^1^, Erick Bandala^1^, Fazlay Rubbi^5^, Guangrui Chai^6^, Zhengyi Zhang^7^, Yiliang Liao^5, *^, Danyang Zhao^2,^ ^*^, Yan Wang^1, *^, Akhilesh K. Gaharwar^8, *^, Yifei Jin^1, *^

**This PDF file includes:**

**Figs. S1** to **S16**

**Tables S1** to **S8**

**Other Supporting Information for this manuscript include the following:**

**Movies S1** to **S9**

**Supporting Information S1**. Visualization of nanoclay-hydrogel nanocomposites with different formulas


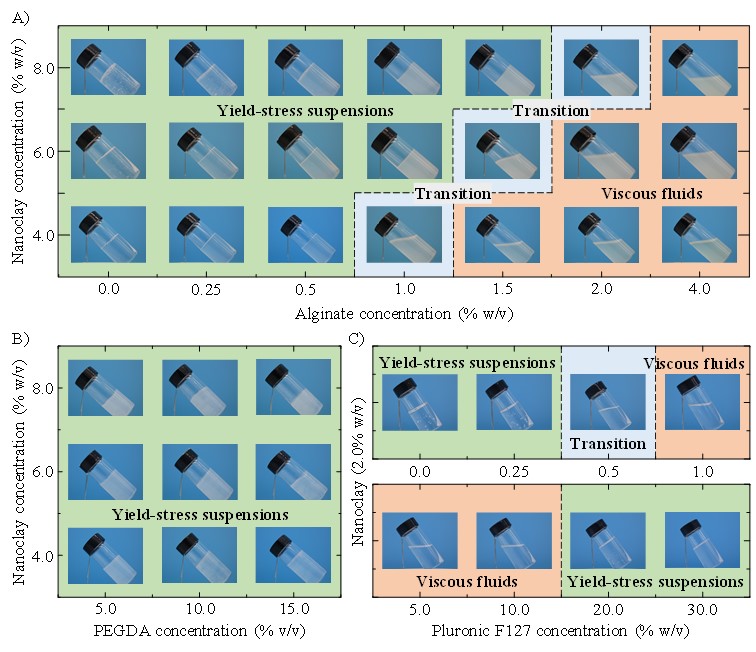


**Figure S1.** Visualization of **A)** nanoclay-alginate, **B)** nanoclay-PEGDA, and **C)** nanoclay-Pluronic F127 nanocomposites with different formulas.

**Supporting Information S2**. Rheology of nanoclay-alginate nanocomposites

**Figure S2.** Shear stress-shear rate curves of nanoclay-alginate nanocomposites with **A)** 4.0%, **B)** 6.0%, and **C)** 8.0% (w/v) nanoclay.

**Supporting Information S3**. SEM images and EDS analyses of nanoclay-alginate nanocomposites

**Figure S3.** SEM images and element distributions of 6.0% (w/v) nanoclay with **A)** 0.5%, **B)** 1.0%, **C)** 1.5%, and **D)** 4.0% (w/v) alginate. Scale bars: 20 µm.

**Supporting InformationS4**. SANS data of nanoclay-alginate nanocomposites

**Figure S4.** SANS data of 6.0% (w/v) nanoclay with **A)** 0.25% and 0.5% (w/v) alginate, **B)** 1.5% and 2.0% (w/v) alginate, and **C)** 4.0% (w/v) alginate. **D)** SANS scale characterization of nanoclay-alginate system.

**Table S1.** Parameters for fitting the SANS data of nanoclay-alginate nanocomposites.

| Parameters | Values | References | Target parameters |
| --- | --- | --- | --- |
| *scale_particle_* (6%) | 0.02268 | [S1] | *C*, *A*, *ξ* |
| *R* (6%) | 85.48±0.23/*Å* | Experiments |  |
| *L* (6%) | 10.33±0.87/*Å* |  |  |
| *ρ_p_* | 4.18×10-6/*Å^-2^* | [S2] |  |
| *ρ_s_* | 6.38×10-6/*Å^-2^* |  |  |

**Supporting Information S5**. Rheology of nanoclay-PEGDA nanocomposites

**Figure S5**. Shear stress-shear rate curves of nanoclay-PEGDA nanocomposites with **A)** 4.0%, **B)** 6.0%, and **C)** 8.0% (w/v) nanoclay.

**Supporting Information S6**. SEM images of nanoclay-PEGDA nanocomposites

**Figure S6.** SEM images of nanoclay-PEGDA nanocomposites with different formulas. Scale bars: 20 µm.

**Supporting Information S7**. EDS analysis of nanoclay-PEGDA nanocomposites

**Figure S7.** EDS analysis results of nanoclay-PEGDA nanocomposites with different formulas (carbon atom in red and silicon atom in blue). Scale bars: 20 µm.

**Supporting Information S8**. Element distribution in nanoclay-PEGDA nanocomposites

**Figure S8.** Area fraction of carbon and silicon atoms as a function of PEGDA concentration.

**Supporting Information S9**. SANS data of nanoclay-PEGDA nanocomposites

**Figure S9.** SANS data of nanoclay-PEGDA nanocomposites with different formulas.

**Table S2.** Parameters for fitting the SANS data of nanoclay-PEGDA nanocomposites.

| Parameters | Values | References | Target parameters |
| --- | --- | --- | --- |
| *scale_particle_* (4%) | 0.01512 | [S1] | *C*, *A*, *ξ* |
| *scale_particle_* (6%) | 0.02268 |  |  |
| *scale_particle_* (8%) | 0.03024 |  |  |
| *R* (4%) | 95.95±0.32/*Å* | Experiments |  |
| *R* (6%) | 85.48±0.23/*Å* |  |  |
| *R* (8%) | 67.40±0.14/*Å* |  |  |
| *L* (4%) | 10.12±1.72/*Å* |  |  |
| *L* (6%) | 10.33±0.87/*Å* |  |  |
| *L* (8%) | 9.69±0.81/*Å* |  |  |
| *ρ_p_* | 4.18×10-6/*Å*^-2^ | [S2] |  |
| *ρ_s_* | 6.38×10-6/*Å*^-2^ |  |  |

**Supporting Information S10.** Correlation length in the nanocomposites at different PEGDA concentrations

**Figure S10.** Correlation length in the nanocomposites as a function of PEGDA concentration.

**Supporting Information S11**. Rheology of nanoclay-Pluronic F127 nanocomposites

**Figure S11.** Yield stresses of the nanoclay-Pluronic F127 nanocomposites with different formulas.

**Supporting Information S12**. SEM images of nanoclay-Pluronic F127 nanocomposites

**Figure S12.** SEM images of nanoclay-Pluronic F127 nanocomposites with different formulas. Scale bars: 400 nm.

**Supporting Information S13**. EDS analysis of nanoclay-Pluronic F127 nanocomposites

**Figure S13.** EDS analysis results of nanoclay-Pluronic F127 nanocomposites with different formulas (carbon atom in red and silicon atom in blue). Scale bars: 400 nm.

**Supporting Information S14**. SANS data of nanoclay-Pluronic F127 nanocomposites

**Figure S14.** SANS data of nanoclay-Pluronic F127 nanocomposites with different formulas.

**Table S3.** Parameters for fitting the SANS data of nanoclay-Pluronic F127 nanocomposites (core-shell).

| Parameters | Values | References | Target parameters |
| --- | --- | --- | --- |
| *scale_particle_* (2%) | 0.00756 | [S1] | *t_F_*, *t_R_* |
| *R* (2%) | 132.12±0.87/*Å* | Experiments |  |
| *L* (2%) | 10.39±0.23/*Å* |  |  |
| *V_p_* | 569773.89/*Å^3^* |  |  |
| *ρ_p_* | 4.18×10-6/*Å^-2^* | [S2] |  |
| *ρ_s_* | 6.38×10-6/*Å^-2^* | [S3] |  |

**Table S4.** Parameters for fitting the SANS data of nanoclay-Pluronic F127 nanocomposites (FCC or BCC).

| Parameters | Values | References | Target parameters |
| --- | --- | --- | --- |
| *scale_m_* (20%) | 0.1904 | [S1, S3] | *d_n_, r_m_* |
| *scale_m_* (30%) | 0.2857 |  |  |
| *ρ_PF127_* | 0.52×10-6/*Å^-2^* | [S1] |  |
| *ρ_s_* | 6.38×10-6/*Å^-2^* | [S2] |  |

**Supporting Information S15**. SANS data and particle sizes of pure nanoclay suspensions

**Figure S15.** **A)** SANS data and **B)** systematic particle sizes of pure nanoclay suspensions.

**Table S5.** Parameters for fitting the SANS data of pure nanoclay suspensions.

| Parameters | Values | References | Target parameters |
| --- | --- | --- | --- |
| *scale_particle_* (2%) | 0.00756 | [S1] | *R*, *L* |
| *scale_particle_* (4%) | 0.01512 |  |  |
| *scale_particle_* (6%) | 0.02268 |  |  |
| *scale_particle_* (8%) | 0.03024 |  |  |
| *ρ_p_* | 4.18×10-6/*Å^-2^* | [S2] |  |
| *ρ_s_* | 6.38×10-6/*Å^-2^* |  |  |

**Supporting Information S16**. Liquid baths and ink for filament printing

**Table S6.** Liquid baths and ink for filament printing.

| Liquid bath | Yield stress (Pa) | Thixotropic response time (s) | Ink formula | Printing speed (mm/s) | Filament type |
| --- | --- | --- | --- | --- | --- |
| 2% nanoclay-30% Pluronic F127 | 414.9 | 0.46 | 7% NaAlg-15% PEGDA-0.1% photo-initiator | 110 | Stretched filament |
| 8% nanoclay-10% PEGDA | 350.6 | 0.07 |  |  | Broken filament |
| 6% nanoclay-1% NaAlg | 174.8 | 0.27 |  |  | Stretched filament |
| 6% nanoclay-10% PEGDA | 175.3 | 0.12 |  |  | Broken filament |
| 6% nanoclay-1.5% NaAlg | 17.2 | 0.21 |  |  | Discontinuous filament |
| 2% nanoclay-1% Pluronic F127 | 0 | 0.09 |  |  | Sinking filament |

**Supporting Information S17**. Shape fidelity of printed human kidney analog

**Figure S16.** Overall dimensions of the printed anatomic-size human kidney model.

**Table S7.** Relative errors of the overall dimensions of the printed anatomic-size human kidney analog.

| Key dimension | Designed value (mm) | Measured value  (mm) | Relative error  (%) |
| --- | --- | --- | --- |
| *L_1_* | 98.65 | 100.43 ± 1.26 | 1.80 |
| *L_2_* | 43.47 | 44.26 ± 0.41 | 1.83 |
| *L_3_* | 36.88 | 38.28 ± 0.59 | 3.79 |

**Supporting Information S18**. Comparison of high-speed EIW with other 3D bioprinting methods

**Table S8.** Comparison of high-speed EIW with other 3D bioprinting methods.

| Method | Printing speed | Multiple material printing | Material requirement | Scaled-up structure | Reference |
| --- | --- | --- | --- | --- | --- |
| Inkjet bioprinting | High | Easy | Low viscosity | Difficult | [S4-S7] |
| Digital light processing | High | Difficult | Photocurable | Easy |  |
| Stereolithography | Low | Difficult | Photocurable | Medium |  |
| Direct ink writing | Medium | Easy | High viscosity | Difficult |  |
| Current EIW | Low | Easy | N/A | Easy |  |
| High-speed EIW | High | Easy | N/A | Easy | N/A |

**References**

[S1] A. Nelson, T. Cosgrove, Small-angle neutron scattering study of adsorbed Pluronic tri-block copolymers on Laponite. *Langmuir* **2005**, *21(20)*, 9176-9182.

[S2] A. Nelson, T. Cosgrove, A small-angle neutron scattering study of adsorbed poly(ethylene oxide) on Laponite. *Langmuir* **2004**, *20(6)*, 2298-2304.

[S3] D. C. Pozzo, L. M. Walker, Small-angle neutron scattering of silica nanoparticles templated in PEO–PPO–PEO cubic crystals. *Colloids. Surf. A Physicochem. Eng. Asp.* **2007**, *294(1-3)*, 117-129.

[S4] S. V. Murphy, A. Atala, A. 3D bioprinting of tissues and organs. *Nat. Biotechnol.* **2014**, *32(8)*, 773-785.

[S5] S. Agarwal, S. Saha, V. K. Balla, A. Pal, A. Barui, S. Bodhak, Current developments in 3D bioprinting for tissue and organ regeneration–a review. *Front. Mech. Eng.* **2020**, *6*, 589171.

[S6] S. Vanaei, M. S. Parizi, F. Salemizadehparizi, H. R. Vanaei, An overview on materials and techniques in 3D bioprinting toward biomedical application. *Eng. Regen.* **2021**, *2*, 1-18.

[S7] M. Mirshafiei, H. Rashedi, F. Yazdian, A. Rahdar, F. Baino, Advancements in tissue and organ 3D bioprinting: Current techniques, applications, and future perspectives. *Mater. Des.* **2024**, *240*, 112853.
